# Case report: 5-year progression free survival and complete liver response in a patient with metastatic breast cancer treated with everolimus plus exemestane

**DOI:** 10.1097/MD.0000000000021211

**Published:** 2020-07-31

**Authors:** Eriseld Krasniqi, Giacomo Barchiesi, Marco Mazzotta, Laura Pizzuti, Alice Villa, Maddalena Barba, Patrizia Vici

**Affiliations:** a Division of Medical Oncology 2, IRCCS Regina Elena National Cancer Institute, Rome; b UOC Oncologia, Ospedale dell’Angelo, Mestre; c Endocrinology Unit, Fondazione Policlinico Universitario A. Gemelli IRCCS, Roma – Università Cattolica del Sacro Cuore, Italy.

**Keywords:** everolimus, exemestane, metastatic breast cancer

## Abstract

**Rationale:**

Within a rapidly expanding therapeutic armamentarium, the combination of everolimus (Eve) plus exemestane (Exe) utility needs to be reinstated in hormone receptor positive (HR+), human epidermal growth factor receptor 2 negative (HER2–) metastatic breast cancer (MBC).

**Patient concerns:**

We herein report on a patient affected by HR+ HER2– MBC treated with radical surgery after neoadjuvant chemotherapy, who relapsed early on adjuvant tamoxifen, progressed rapidly on first line anastrozole, and failed treatment with third line capecitabine.

**Diagnoses:**

Metastatic luminal breast cancer progressed under standard endocrine therapy and chemotherapy.

**Interventions:**

Third line with Eve plus Exe was given after chemotherapy.

**Outcomes:**

Patient experienced a 5-year progression free interval.

**Lessons:**

Eve plus Exe remains a valid option in HR+HER2– MBC.

## 1 Introduction

Endocrine therapy (ET) represents the mainstay of treatment in hormone receptor positive, human epidermal growth factor receptor 2 negative metastatic breast cancer (HR+HER2– MBC). The proved efficacy and safety profile of endocrine agents encourage their use in the metastatic setting, unless the occurrence of visceral crisis.^[1]^ Unfortunately, all patients will experience disease progression due to endocrine resistance.^[2]^ Evidence supports the synergism between the mammalian target of rapamycin (mTOR) inhibitors and ET in terms of antitumoral activity.^[3]^ Everolimus (Eve) inhibits the mTOR pathway leading to apoptosis, restraining of cell growth and proliferation. Eve in combination with exemestane (Exe) was approved in 2012 based on the results of the BOLERO-2 trial.^[4]^ We herein report on the exceptional outcome of a 48-year-old female breast cancer patient treated with Eve plus Exe in 3rd line.

## 2 Case presentation

**Figure 1:**
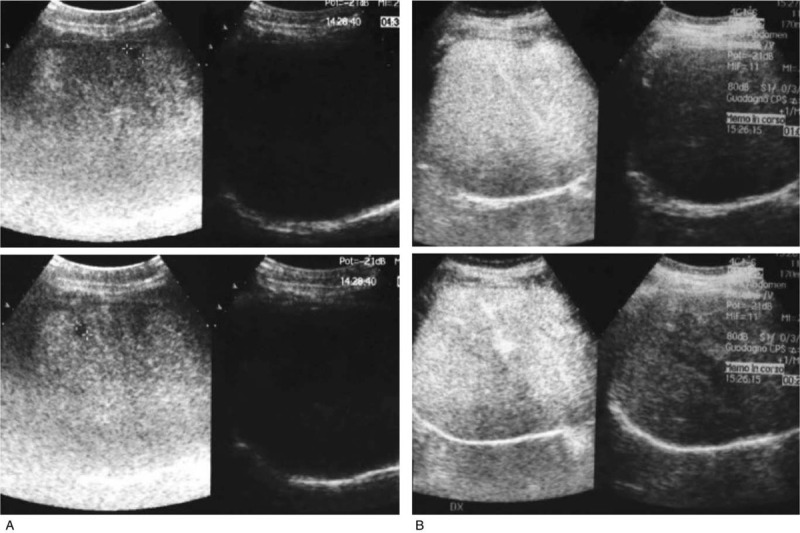
Liver ultrasound before (A) and after (B) 39 months of everolimus plus exemestane.

In April 2010, our patient, a 48-year-old, premenopausal, Caucasian woman identified a mass in her right breast. The physical examination showed an about 4 cm lesion in the retro-areolar region adherent to the superficial skin layer, which appeared intact, and abnormal lymph nodes of about 1 cm. The mammogram revealed increased microcalcifications, skin thickening, and nipple retraction and a subsequent ultrasound showed ductal ectasias with sporadic hyperechoic spots. An ultrasound-guided biopsy was performed on the right breast mass and on the ipsilateral lymph nodes. The pathological examination identified a lobular invasive carcinoma in both the breast and ipsilateral axillary nodes. The biological characterization was as follows: estrogen receptor 60%, progesterone receptor 80%, HER2 negative (–), Ki-67 10%. No evidence of metastatic spread appeared at basal staging (chest X-ray, abdominal ultrasound and bone scintigraphy were all negative), which resulted into clinical IIB (cT2cN1(f)cM0) according to the AJCC TNM staging system 7^th^ edition. Given the pre-operative size of the primary tumour, that is 4 cm in its larger diameter, and the histologically confirmed involvement of the ipsilateral axillary lymph nodes, from May 2010 through October 2010, this patient received standard neoadjuvant chemotherapy with epirubicin/cyclophosfamide followed by docetaxel. In November 2010, she underwent bilateral mastectomy and right lymph node dissection. The pathological report described a minimal invasive disease (1 mm) in the right breast. The biologic features were estrogen receptor / progesterone receptor%: 20/0, HER2–, Ki-67 20%. The final pathologic stage was ypT1ypN0 (0/12). No carcinoma was found in the left breast. The patient started adjuvant tamoxifen in January 2011, which was interrupted in May 2012, when an annual follow-up bone scan performed in April 2012 showed bone metastasis at the left sacroiliac joint. Restaging was completed in May 2012 by a chest X-ray and abdominal ultrasound that showed no evidence of disease. In this same month, circulating levels of biomarkers were as it follows: carcinoembryonic antigen: 9.8 μg/L and CA 15-3: 36 U/mL. The low disease burden with no visceral localizations and the switch to the postmenopausal status oriented toward a first line treatment with anastrozole (Anas). The patient underwent also treatment with i.v. zoledronic acid 4 mg every 28 days from June 2012 to November 2012, when the agent was suspended for a dental intervention. In September 2012, the disease further progressed at the bone level as showed by a bone scan, and liver metastases appeared in the ultrasound. No CT scan images are available for further documenting liver progression. No abnormalities emerged from the chest X-ray. The patient received capecitabine (Cape) in second-line. Palliative radiation of the cervical column (30 gray) and right hip bone (40 gray) was administered. In February 2013, revaluation showed bone and liver progression of disease (PD). A third line treatment with Eve (10 mg once daily) plus Exe (25 mg once daily) was initiated. The disease started to respond after 4 months of treatment and the best response was obtained after 39 months of treatment reaching complete response of the liver disease (Fig. 1), while bone metastases remained stable. The total duration of response was 35 months. Eve dosage was reduced to 5 mg due to a grade 3 mucositis. This treatment continued until March 2018, when a CT-scan documented lung and bone PD. Overall, our patient experienced a 5-year progression free survival (PFS) on Eve plus Exe treatment, with an acceptable quality of life and an eastern cooperative oncology group performance status of 0. Afterwards the patient was treated with fulvestrant (Fulv) (500 mg i.m. on days 1, 15, 29, and monthly thereafter) plus palbociclib (125 mg die for 21 days, followed by 7 days off) from April 2018 to August 2018, when she experienced further liver PD. The last line of treatment consisted in eribuline (1.3 mg / m^2^ i.v. in days 1 and 8 of a 21-day cycle), which was started in September 2018 and stopped in August 2019 because of further liver PD and worsening of the eastern cooperative oncology group status. The patient died in October 2019. In this study we used The Response Evaluation Criteria in Solid Tumors version 1.1 (RECIST v. 1.1) to measure the disease response to treatments and Common Terminology Criteria for Adverse Events version 4.0 to quantify the emerged adverse events related to the mentioned drugs.

## 3 Discussion

In the case we presented, the disease could be deemed as primarily resistant following the early failure of 2 ET lines. The patient was not experiencing a visceral crisis, thus among the 2 remaining options for the second-line consisting in chemotherapy or Fulv, we preferred Cape. When the patient had a PD on Cape, in February 2013, she presented with liver and bone metastases. By that time, besides the treatment with Fulv, also the treatment with Eve plus Exe had become a viable option in this setting of patients based on the BOLERO-2 trial,^[4]^ which enrolled postmenopausal women affected by HR+/HER2– MBC refractory to non-steroidal aromatase inhibitors and randomized them to Exe versus Eve plus Exe. Results showed that the addition of Eve increased median PFS from 2.8 to 6.9 months, with benefit being registered also in patients with visceral disease and who had received 3 or more previous lines of treatment. On the other hand, the best evidence regarding Fulv for the setting of our patient was available from the SWOG randomized trial, which compared Fulv plus Anas versus Anas alone in the first-line for HR+/HER2– MBC.^[5]^ Compared to Anas, the combination with Fulv improved PFS (13.5 vs 15.0 months) and OS (41.3 vs 47.7 months). However, the enrolled patients had not received previous treatments for metastatic disease and very few of them presented AI resistance from the adjuvant setting. Based on these data, we judged the Eve plus Exe combination as most suitable for our patient at that time and as the most cost-effective option, even though we were aware of the approximately 10 times higher monthly cost of the Eve plus Exe combination compared to Fulv alone.

The characterization of patient's subsets who may most benefit from the use of a given therapeutic agent is of utmost importance. A wide range of clinical-pathologic features and an even greater pool of biomarkers assessed in biological samples throughout the most groundbreaking “omics’-related platforms may help reach this goal. We characterized 102 postmenopausal HR+HER2– patients for relevant anthropometric and metabolic parameters, which may affect response to eve plus exe, with some interesting results, which are still to be confirmed.^[6]^ In the case presented, we did not assess any specific biomarker, neither we identified any clinical-pathological feature which may provide an explanation to our singular finding. Rapidly accumulating evidence suggests that the answer to ours and similar questions may have molecular roots. The genetic landscape of tumors from patients enrolled in the BOLERO-2 trial showed that PIK3CA mutations affected only minimally the efficacy of Eve, while mTOR mutations and chromosomal instability seemed to show greater impact.^[7,8]^

Given the outcome observed, we conclude that our patient may have deserved a genetic assessment, possibly integrated by transcriptomic profiling. More generally, investigating the molecular characteristics of patients with uncommon treatment outcomes may provide precious clues on the underlying biological mechanisms, and, at the individual patient level, may help allocate the next to come therapeutic choice within a personalized therapeutic continuum.

## Acknowledgments

We thank Doctor Rosa Carbone and Ana Maria Edlisca for administrative support.

## Author contributions

**Conceptualization:** Eriseld Krasniqi, Patrizia Vici.

**Data curation:** Giacomo Barchiesi.

**Formal analysis:** Eriseld Krasniqi.

**Supervision:** Eriseld Krasniqi, Laura Pizzuti, Maddalena Barba, Patrizia Vici.

**Validation:** Eriseld Krasniqi.

**Visualization:** Giacomo Barchiesi, Marco Mazzotta.

**Writing – original draft:** Eriseld Krasniqi, Alice Villa, Maddalena Barba.

**Writing – review & editing:** Barchiesi Giacomo, Marco Mazzotta, Laura Pizzuti, Maddalena Barba, Patrizia Vici.
